# 高通量蛋白质组学分析研究进展

**DOI:** 10.3724/SP.J.1123.2020.08023

**Published:** 2021-02-08

**Authors:** Qiong WU, Xintong SUI, Ruijun TIAN

**Affiliations:** 南方科技大学理学院化学系, 广东 深圳 518055; Department of Chemistry, School of Science, Southern University of Science and Technology, Shenzhen 518055, China; 南方科技大学理学院化学系, 广东 深圳 518055; Department of Chemistry, School of Science, Southern University of Science and Technology, Shenzhen 518055, China; 南方科技大学理学院化学系, 广东 深圳 518055; Department of Chemistry, School of Science, Southern University of Science and Technology, Shenzhen 518055, China

**Keywords:** 高通量, 蛋白质组学, 质谱, 色谱, 样品前处理, high-throughput, proteomics, mass spectrometry (MS), chromatography, sample preparation

## Abstract

基于质谱的蛋白质组学技术已经日趋成熟,可以对细胞和组织中的成千上万种蛋白质进行全面的定性和定量分析,逐步实现“深度覆盖”。随着生物医学日益增长的大队列蛋白质组学分析需求,如何在保持较为理想的覆盖深度下实现短时间、快速的“高通量”蛋白质组学分析已成为当前亟需解决的关键问题之一。常规的蛋白质组学分析流程通常包括样品前处理、色谱分离、质谱检测和数据分析。该文从以上4个方面展开介绍近10年以来高通量蛋白质组学分析技术取得的一系列研究进展,主要包括:(1)基于高通量、自动化移液工作站的蛋白质组样品前处理方法;(2)基于微升流速液相色谱与质谱联用的高通量蛋白质组检测方法;(3)利用灵敏度高、扫描速度快的质谱仪实现短色谱梯度分离下蛋白质组深度覆盖的分析方法;(4)基于人工智能、深度神经网络、机器学习等的蛋白质组学大数据分析方法。此外,对高通量蛋白质组学面临的挑战及其发展进行展望。总而言之,预期在不久的将来高通量蛋白质组学技术将会逐步“落地转化”,成为大队列蛋白质组学分析的利器。

## Abstract (en)

Proteomic analysis aims at characterizing proteins on a large scale, including their relative abundance, post-translational modifications, protein-protein interactions and so on. Proteomic profiling helps to elucidate the mechanisms of disease occurrence and to discover new diagnostic markers and therapeutic targets. Mass spectrometry (MS)-based proteomic technologies have advanced to allow comprehensive qualitative and quantitative proteome profiling across a myriad proteins in cells and tissues. High-throughput proteomics is the core technique for large-scale protein characterization. With the increased demand for large cohort proteomic analysis in the biomedical research field, high-throughput proteomic analysis has become a critical issue that needs to be urgently addressed. The standard shotgun proteomic workflow comprises four steps, including sample preparation, peptide separation, MS acquisition, and data analysis. Advances in these four steps have contributed to the development of high-throughput proteomics. In this review, we aimed at summarizing the current information on the state-of-the-art development of high-throughput proteomic analysis, mainly including the following topics: (1) High-throughput, automatic proteomic sample preparation methods based on liquid-handling workstations. The automation of the proteomic sample preparation steps is essential for high-throughput proteomic analysis, which will significantly reduce variation of manual operation and sample loss by multistep sample processing. The commercial liquid handling workstations, including King Fisher^TM^ Flex, Agilent Bravo, AssayMAP Bravo, and Biomek^®^ NX^P^, perform the handling steps of 96- or 384-channel microplate formats using a mechanical arm that increases the throughput and robustness of sample preparation. (2) High-throughput proteomic detection methods based on microliter-flow-rate liquid chromatography coupled with mass spectrometry (micro-flow LC-MS/MS). Nanoliter-flow-rate liquid chromatography coupled with mass spectrometry (Nano-flow LC-MS/MS) is widely used in classic proteomic research due to its excellent sensitivity, which often comes at the expense of robustness. Owing to the improved robustness and decreased injection-to-injection overheads, micro-flow LC-MS/MS has become increasingly popular in high-throughput proteomic analysis. (3) Using MS instrumentation with high sensitivity and fast scanning speed to realize in-depth proteomic analysis coupled with short chromatographic gradient separation. In recent years, new MS instrumentation continues to exhibit speed of analysis and sensitivity enables the large-scale profiling of hundreds of samples. In particular, ion mobility-based MS, such as timsTOF Pro and Exploris 480 equipped with a front-end high field asymmetric waveform ion mobility spectrometry (FAIMS), which provides fast, sensitive, and robust proteome profiling, thus shifting proteomics to the high-throughput era. (4) Artificial intelligence-, deep neural network-, and machine learning-based proteome data analysis methods. These approaches have improved comprehensive proteomic analysis efficiency. Specifically, the emergence of new algorithms and the up gradation of search engines accelerate the process of high-throughput data analysis. Additionally, the challenges and future development of high-throughput proteomics are prospected. In conclusion, high-throughput proteomic technologies are expected to gradually “transform” and become powerful tools for large cohort proteomic analysis in the near future.

蛋白质组学是指大规模地对蛋白质的表达水平、翻译后修饰、蛋白质相互作用等进行研究。蛋白质组研究不仅可以全景式地揭示生命活动的分子本质,还能阐明生命在生理或病理条件下的变化机制^[1]^。近年来,色谱和质谱技术的进步驱动了蛋白质组学的快速增长,逐步实现了“深度覆盖”,科学家们先后完成包括人类在内的多个物种的蛋白质组图谱解析^[2,3]^。2020年,Mann等^[4]^系统性地绘制了100个跨物种的蛋白质组图谱,获得了200万条肽段和34万个蛋白质的鉴定信息,为整个进化范围内生物的功能组织研究提供重要依据。随着生物医学研究日益增长的大队列蛋白质组学分析需求,如何实现“高通量”的蛋白质组学分析已成为当前亟需解决的关键问题之一。常规的蛋白质组学分析流程通常包括样品前处理、色谱分离、质谱检测和数据分析。该文将从以上4个方面介绍近10年以来高通量蛋白质组学分析技术取得的相关研究进展。

## 1 样品前处理

样品前处理是整个蛋白质组学分析流程的关键步骤,传统样品前处理流程依赖于多步的手工操作,不仅耗时且不可避免地存在人为误差,成为限制蛋白质组学发展的瓶颈之一。集成化的样品前处理技术有利于提升蛋白质组学分析的整体性能和灵敏度,如大连化物所张丽华课题组^[5]^结合在线蛋白质酶解,同位素二甲基化标记和多维肽段分离发展了蛋白质组定量集成化平台,具有灵敏度高、定量重现好等优点。本课题组前期开发了一种基于离心力的集成化蛋白质组学样品前处理技术(simple and integrated spintip-based proteomics technology, SISPROT)^[6]^,通过将强阳离子交换填料和C18膜填充在枪头中以集成化方式完成样品前处理和多肽分级的全过程,显著提升了蛋白质组学分析的整体性能和灵敏度,并得到进一步推广^[7,8,9,10,11]^。高通量蛋白质组学则对样品前处理提出了更高要求,发展分析通量高、快速而稳定的样品前处理方法成为这一领域亟需解决的首要问题。2014年,Yu等^[12]^在超滤膜辅助的样品前处理方法(filter-aided sample preparation, FASP)基础上发展了96通道的FASP(96FASP),该方法借助于96孔滤膜板进行离心操作,从10 μg临床尿液中可鉴定700~900个蛋白质。Mann课题组^[13]^则发展了in-StageTip(iST)技术,在内置C18膜的封闭Tip头内可完成样品前处理,并开发了PreOmics试剂盒(https://preomics.com/products)。通过进一步定制96通道的iST设备(96-well iST)并借助于多通道移液器可完成96个样品前处理操作。然而,以上的样本前处理方法虽然提高了分析通量,但仍然依赖于手工移液和转移操作。

近十几年来,自动化移液工作站相继问世,如赛默飞的自动化磁珠提取系统King Fisher^TM^ Flex、安捷伦高精度的Bravo和精确流速控制的AssayMAPBravo、贝尔曼库尔特的Biomek^®^ NX^P^自动化工作站等,使样品前处理逐渐实现自动化。自动化移液工作站借助于机械臂完成96甚至384通道的移液操作,解放手工操作,大大促进了高通量的样品前处理技术发展。Mann等^[14]^将iST技术与Bravo自动化平台联用,2 h内可完成96个血浆样品的前处理。最近,他们还将这项前处理工作流程应用于100个不同类别生物体的蛋白质图谱解析^[4]^。Krijgsveld等开发了基于磁珠的样品前处理技术(single-pot solid-phase-enhanced sample preparation, SP3)^[15,16]^,进一步将SP3技术与Bravo平台相结合开发了autoSP3技术,3.5 h可完成96个样品的前处理,并具有优异的稳定性^[17]^。Leutert等^[18]^发展了快速,自动化的磷酸化蛋白质组学分析流程(rapid-robotic phosphoproteomics, R2-P2),在King Fisher^TM^ Flex自动化工作站上整合了SP3技术和基于Fe-IMAC磁珠的磷酸化富集技术,实现5 h的磷酸化蛋白质组前处理。自动化移液工作站保证高通量分析的同时,还能极大缩短前处理时间,以及减少大队列样品处理造成的人为误差。然而,以上的这些样品前处理工作流程还未能实现“零人工”操作的全自动化。随着高通量样品前处理的不断智能化,开发“零人工”操作的全自动化样品前处理方法成为必然的发展趋势。

## 2 色谱分离

色谱分离是液相色谱-质谱联用蛋白质组学分析中的关键环节^[19,20,21]^,但目前的液相色谱-质谱联用技术存在液相分离速度难以匹配质谱采集速度、稳定性不够好等问题^[22]^,限制了高通量蛋白质组学的进一步发展。因此,在保证较为理想的蛋白质组覆盖度前提下,缩短液相色谱分离时间并提高分离稳定性以满足高通量数据采集的需求显得尤为重要。传统的“鸟枪法”蛋白质组学研究一般选择纳升级液相来获得更高的检测灵敏度,与此同时却牺牲了分析通量与液相色谱分离的稳定性。近年来,微升流速液相色谱(micro-flow LC)或高流速液相色谱(high-flow LC)因其色谱稳定性好、周转时间短等优点广泛应用在高通量蛋白质组学。通过配合使用先进的高灵敏度质谱仪,可以有效解决由于稀释效应导致的灵敏度下降问题。本课题组在前期开发的Photo-pTyr-scaffold^[23]^与基于96孔板的Photo-pTyr-scaffold正向蛋白阵列平台^[24]^的基础上,开发了高通量、快速的酪氨酸磷酸化(pTyr)信号复合物的蛋白质组学分析工作流程^[25]^。利用150 mm×150 μm分离柱在分离流速800 nL/min和20 min有效色谱梯度条件下完成高通量蛋白质组学分析;通过与Q Exactive HF-X质谱仪联用,在不增加样品上样量和不影响鉴定覆盖度前提下,能够快速、可重复地分析动态pTyr信号蛋白质复合物^[25]^。Bruderer等^[26]^利用150 mm×300 μm分离柱(流速5 μL/min,梯度40 min)完成了1508例血浆样本的分析,并且超过2000次的进样没有发现堵塞(背压只增加14%),表明micro-flow LC具有良好的稳定性。Bian等^[27]^利用商品化150 mm×1 mm的C18色谱分离柱,以50 μL/min微升级流速联合Q Exactive HF-X质谱仪进行分析。在2000个蛋白质组的分析中具有色谱保留时间(相对标准偏差<0.3%)和蛋白质定量(相对标准偏差<7.5%)的优异重现性,并在16 h内完成9000多种蛋白质的深度覆盖分析;当分析超过7500个样品时,micro-flow LC-MS/MS的性能并没有明显下降。在COVID-19新冠病毒的临床蛋白质组学研究中,Messner等^[28]^将Agilent 1290 Infinity II与TripleTOF 6600联用,利用5 min的有效色谱梯度分离(50 mm×2.1 mm分离柱,流速800 μL/min)可实现每天180个样品的分离分析。以上研究结果表明,micro-flow LC和high-flow LC具有分析通量高、稳定性好等优点,在高通量蛋白质组学分析中将具有广阔的应用前景。

另外一方面,蛋白质组检测效率受限于传统液相色谱系统的设计和分离能力,难以突破真正意义上的高通量分析。为了克服这一不足,新的液相系统应运而生。2018年,丹麦Evosep公司推出的新型液相系统-Evosep One同时具备创新型高低压管路系统设计以及样品洗脱和梯度预存储技术,可实现同步的快速上样和色谱梯度分离,大幅度减少液相色谱分析时间。此外,Evotip技术通过减少色谱柱堵塞和残留,提高了系统的稳定性^[29]^。Mann等^[30]^利用Evosep One与timsTOF Pro质谱仪联用,实现每天200个样本分析(5.6 min有效梯度),在16次重复中鉴定到1231种蛋白质(进样量为50 ng)。因此,Evosep One能够极大地提高样本分析通量且具有优异的稳定性,从而助力蛋白质组学分析真正跨入高通量时代。此外,Evosep one搭载Q Exactive HF-X/HF^[31]^、Orbitrap Fusion Lumos^[32,33]^、Orbitrap Exploris 480^[34]^等质谱在不同大队列蛋白质组样品中也得到了逐步推广。

## 3 质谱检测

质谱仪是蛋白质组学的检测利器,质谱仪硬件和技术上的革新一直引领着蛋白质组学技术的高速发展。为了应对高通量蛋白质组学挑战,开发扫描速度快,灵敏度高的质谱仪是近年来质谱硬件发展的趋势。近年来,质谱仪硬件方面的重大突破在于离子淌度功能(ion mobility)的引入,为蛋白质组学分析增加了全新的分离维度,从而获得更加全面的鉴定信息^[35,36]^。布鲁克公司开发的timsTOF Pro质谱采用了捕集型离子淌度(trapped ion mobility spectrometry, TIMS)和平行累积连续碎裂(parallel accumulation serial fragmentation, PASEF^[30]^)采集技术,将离子累积、母离子选择和碎裂同步进行,从而实现了近100%的离子利用率、接近120 Hz二级谱扫描速度和超过20倍灵敏度的提高^[30]^。Mann等^[29]^通过将timsTOF Pro与Evosep One联用,在11.5 min有效梯度下(每天100个样本),从重症感染患者的血浆中(上样量为100 ng)定量到500个蛋白质,表明timsTOF Pro拥有极高的采集速度与灵敏度。另外,离子淌度的引入可以实现保留时间、质荷比、离子强度和离子淌度的四维数据采集,使得蛋白质组学进入了4D新时代。基于Orbitrap平台的质谱仪一直是蛋白质组学分析的主流技术。自2011年推出Q Exactive系列,2013年推出Orbitrap Tribrid系列和2019年推出Orbitrap Exploris系列质谱以来,其扫描速度、灵敏度和分辨率在不断提高。例如,Q Exactive的扫描速度和分辨率只有12 Hz和14万(@*m/z* 200)^[37]^。而Exploris 480拥有40 Hz的扫描速度和48万的超高分辨率(@*m/z* 200),并搭载了新一代离子迁移分离装置FAIMS(high-field asymmetric waveform ion mobility spectrometer)。此外,全新四极杆质量分析器使仪器的抗污染能力大幅度提升,并提高了离子选取效率。Olsen等^[34]^对Exploris 480联合FAIMS的性能进行了全面的评价,在每天200个样品的分析通量下(5 min有效色谱梯度)能鉴定到1261个蛋白质。因此,Exploris 480在保证蛋白质鉴定覆盖度的同时具备卓越的稳定性和耐用性。timsTOF Pro与Exploris 480等离子淌度质谱所带来的采集速度和灵敏度的大幅提升使得短色谱梯度分离下也能实现蛋白质的深度覆盖分析,为高通量蛋白质鉴定提供强大的技术支撑。

在过去的10年间,质谱采集技术的发展和革新也推动着蛋白质组学研究迈向高通量。传统的数据依赖型(data-dependent acquisition, DDA)采集模式因随机性、数据重现性差等固有缺陷,限制了其在高通量蛋白质组学中的应用。作为当前最热门的质谱技术之一,数据非依赖型(data-independent acquisition, DIA)的质谱采集模式利用全扫描对每个窗口区域中的所有母离子进行碎裂检测,以获得所有碎片离子信息,具有通量高、重现性好、定量准等优点^[38,39,40]^。上述这些数据采集特征对于高通量蛋白质组的定量分析具有显著优势。近年来,利用DIA进行高通量定量蛋白质组学分析的研究呈现快速增长。Ruedi等^[41]^利用DIA蛋白质组学检测手段研究了232例异卵双生和同卵双生个体的血浆样本,阐述了血浆蛋白质组在遗传和非遗传状态下的变化情况。Mann等^[42]^对来自3个队列的197例人群(非阿尔兹海默病组与阿尔兹海默病组)的脑脊液样本进行DIA数据采集,发现40种蛋白质特征分子可以有效预测阿尔兹海默症。随着4D蛋白质组学(4D-Proteomics)的兴起,timsTOF Pro上最新的DIA技术(DIA-PASEF)赋予其4D数据的采集和匹配能力,增加的离子淌度信息相比传统DIA技术更有效降低了数据匹配的假阳性和缺失值,定量准确度更高,更适合高通量的样本采集。

## 4 数据处理

随着样本制备、色谱分离和质谱技术的共同进步,所要面临的是如何对已获得的海量蛋白质组数据进行高效的数据分析。在计算机技术迅猛发展的今天,基于人工智能、深度神经网络、机器学习等大数据分析方法提高了蛋白质组学数据的分析效率。

以DIA采集为例,共碎裂母离子的干扰导致谱图极其复杂,在短色谱梯度分离的复杂样品中干扰进一步放大。Ralser课题组^[43]^开发了利用深度神经网络分析(用于分辨真实信号与噪声)以及新的量化和信号校正策略来处理DIA数据的软件(DIA-NN),提高了传统DIA蛋白质组学的定性定量分析能力,在3 h内可完成364个酵母样本的高通量数据处理。经典的DIA定量分析流程是预先进行DDA谱图库的构建,再利用DDA谱图库从DIA数据中提取目标肽段的信号进行定量分析。为了进一步推动DIA技术的实用化,复旦大学乔亮课题组^[44]^利用深度学习算法开发了从肽段或蛋白质序列构建谱图库的工具DeepDIA,实现了不依赖于DDA的DIA数据直接分析。Spectronaut是DIA数据检索的主流软件^[45]^,随着Spectronaut 14的发布,其包括的directDIA 2.0新算法可直接利用DIA数据构建虚拟谱图库并进行数据库检索。其算法的定性和定量灵敏度接近DDA分级建库的结果,可以省去耗时的DDA建库过程。此外,Spectronaut 14还可以实现结果文件的自由组合,从而支持上万个DIA样品的检索和合并分析,极大地缩短了高通量的DIA数据解析时间。另外,蛋白质组学搜索引擎的更新,加快了数据分析的进程。军事科学院徐平课题组^[46]^利用pFind的开放式搜索对3种不同酶切的大规模数据进行检索分析,从人睾丸组织中鉴定到了124个潜在的缺失蛋白质。另一方面,Cox团队在今年更新的MaxQuant版本中开发了专为4D-Proteomics使用的4D-MBR算法,即增用CCS值(collision cross section,碰撞截面积)这一新维度进行数据对齐,可显著减少缺失值并提高匹配的可信性^[47]^。他们利用208个血浆样本进行分析,结果显示4D-Proteomics技术可大大提高样本LFQ(label free quantification)的数据完整性,且蛋白质定量数目能提高90%。

## 5 结语

高通量蛋白质组学技术发展至今,从样品自动化的高效制备、快速的蛋白质组全景数据采集、到前沿的数据分析都取得了突飞猛进的进步。“全自动化样品前处理”“微升流速液相色谱分离”“高灵敏离子淌度质谱分析”或将成为高通量蛋白质组学未来几年的研究热点。面对复杂的大队列临床样本,高通量蛋白质组学仍面临如下挑战:(1)如何实现高通量、全自动化的样本前处理和液相色谱-质谱联用分析的无缝对接,从而完成样品制备到数据采集的全流程自动化操作;(2)由于临床样本的宽动态范围和高异质性,短梯度下蛋白质组深度覆盖分析仍需要提高,以期发现更多低丰度的功能生物标志物;(3)蛋白质检测灵敏度和定量准确度需要进一步改善以显著区分噪声与低丰度蛋白质。此外,利用离子淌度气相色谱分离代替传统的液相色谱分离以实现超快速的复杂样品分离可能成为该领域未来潜在的发展方向。总而言之,预期在不久的将来高通量蛋白质组学技术将会逐步“落地转化”,成为大队列蛋白质组学分析的利器。
